# Comprehensive nursing intervention improves psychological outcomes, sleep quality, pain, and coping in patients with comorbid depression and thoracolumbar compression fracture

**DOI:** 10.3389/fpsyt.2026.1820844

**Published:** 2026-06-17

**Authors:** Min Qi, Wenjing Wang, Miao Zhang, Haijing Zhang

**Affiliations:** 1 Department of Orthopedics, First Affiliated Hospital of Hebei Medical University, Shijiazhuang, Hebei, China; 2 Mental Health Center, First Hospital of Hebei Medical University, Shijiazhuang, Hebei, China

**Keywords:** comprehensive nursing, coping styles, depression, pain management, thoracolumbar compression fracture

## Abstract

**Background:**

Thoracolumbar compression fractures frequently coexist with depression, creating a self-reinforcing cycle of pain and psychological distress. Conventional nursing care offers limited psychological support for this comorbid population.

**Methods:**

This quasi-experimental, descriptive study with a non-concurrent historical control enrolled 60 patients with comorbid depression and thoracolumbar compression fracture at a tertiary center in China. The control group (n = 30, February 2019 to March 2021) received conventional nursing, while the research group (n = 30, April 2021 to April 2023) received a comprehensive intervention incorporating psychological counseling, environmental optimization, individualized pain management, health education, structured rehabilitation, and daily living support. Outcomes included depression (SDS), anxiety (SAS), sleep quality (PSQI), pain (VAS), coping styles (SCSQ), and nursing satisfaction, assessed at admission and discharge.

**Results:**

Baseline scores were comparable between groups. Post-nursing, the research group showed significantly lower depression (56.83 vs. 70.17, p < 0.001, d = 1.64), anxiety (54.30 vs. 67.50, p < 0.001, d = 1.69), sleep disturbance (7.83 vs. 12.47, p < 0.001, d = 1.70), and pain scores (3.17 vs. 4.93, p < 0.001, d = 1.50). Positive coping was significantly higher (21.53 vs. 16.07, d = 1.35) and negative coping significantly lower (7.83 vs. 11.27, d = 1.36) in the research group (both p < 0.001). Nursing satisfaction was 96.67% versus 66.67% (p = 0.003). All comparisons survived Holm-Bonferroni correction.

**Conclusion:**

Comprehensive nursing significantly improved psychological status, sleep, pain, and coping in patients with comorbid depression and thoracolumbar compression fracture. The non-randomized design and small sample size limit the strength of these conclusions. Larger prospective randomized trials are needed to confirm these findings.

## Introduction

Thoracolumbar compression fractures are acute injuries that can profoundly affect both physical function and psychological well-being. Although surgical or conservative treatment restores local anatomical integrity, the combined stressors of trauma, postoperative pain, and immobility often impose a substantial psychological burden on patients. Without timely supportive care, this burden may impair sleep quality, reduce treatment adherence, and ultimately prolong rehabilitation (1–3).

Depression is a common comorbidity in patients with painful musculoskeletal conditions, and persistent pain has been identified as an important contributing factor (4). When depression coexists with thoracolumbar compression fracture, the interplay between pain and psychological distress can create a self-reinforcing cycle: unmanaged pain exacerbates depressive symptoms, while depression lowers pain thresholds and diminishes motivation for rehabilitation (5, 6). Recognizing this bidirectional relationship is essential for planning effective nursing care.

Conventional nursing for thoracolumbar fracture patients typically focuses on disease-specific interventions such as condition monitoring, medication administration, and health education, with limited attention to patients’ psychological needs (7). For patients with comorbid depression, this approach may be insufficient. Comprehensive nursing, an integrated model encompassing psychological support, cognitive-behavioral guidance, environmental optimization, individualized pain management, and structured rehabilitation, has shown promise in improving outcomes for patients with either fractures or depression individually (8, 9). However, the specific application of comprehensive nursing to patients presenting with both conditions simultaneously has not been investigated.

The present study therefore aimed to evaluate the effects of a comprehensive nursing intervention on psychological state, sleep quality, pain severity, coping styles, and nursing satisfaction in patients with comorbid depression and thoracolumbar compression fracture, with the goal of informing clinical nursing practice for this population. Specifically, the primary objective was to determine whether the comprehensive nursing intervention produced a greater improvement in psychological state, defined as the primary outcome and measured by depression and anxiety scores, than conventional nursing care. The secondary objectives were to compare the two approaches with respect to sleep quality, pain severity, coping styles, and nursing satisfaction.

## Materials and methods

### Study design

This was a quasi-experimental, descriptive study employing a non-concurrent control design with historical controls. Patients treated during an earlier period (February 2019 – March 2021) who received conventional nursing care served as the control group, while patients treated during a subsequent period (April 2021 – April 2023) who received comprehensive nursing intervention served as the research group. This sequential-cohort approach was adopted because implementing both care protocols simultaneously within the same ward was not feasible; the environmental modifications and workflow changes inherent to the comprehensive nursing model would have contaminated the control condition.

This study was approved by the Ethics Committee of The First Hospital of Hebei Medical University (approval no (2024). Research Review No. 129). All enrolled patients and their family members provided written informed consent prior to participation. Because the study population consisted predominantly of older adults recovering from an acute traumatic fracture with comorbid depression, family members were routinely involved in the consent process in keeping with local clinical and cultural practice. Their written consent was obtained in addition to, and not as a substitute for, the patient’s own consent, in order to ensure that decision-making was fully informed and adequately supported throughout the hospitalization. The study was conducted in accordance with the principles of the Declaration of Helsinki. Patient data were anonymized and handled confidentially throughout the study.

### Participants

#### Setting and enrollment

**Figure 1:**
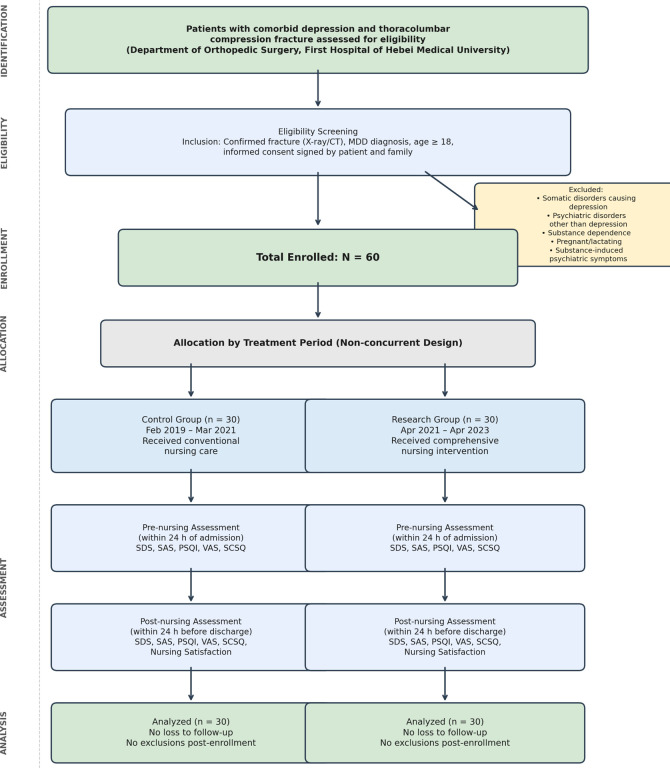
Participant flow diagram showing identification, eligibility screening, group allocation, outcome assessment, and analysis of patients with comorbid depression and thoracolumbar compression fracture.

The study was conducted in the Department of Orthopedic Surgery, The First Hospital of Hebei Medical University, Shijiazhuang, China. The medical records of 30 patients with comorbid depression and thoracolumbar compression fracture treated from February 2019 to March 2021 who received conventional nursing care were reviewed and designated as the control group. Similarly, the records of 30 patients with the same dual diagnosis treated from April 2021 to April 2023 who received comprehensive nursing intervention were reviewed and designated as the research group. The participant flow is illustrated in **Figure 1**. Within each enrollment window, eligible patients were identified through consecutive screening of all admissions to the unit, and every patient who satisfied the inclusion criteria and provided informed consent was enrolled; participant selection was therefore consecutive rather than based on convenience or random sampling. No eligible patient declined participation, no participants were lost to follow-up, and no patients were excluded after enrollment, because all outcomes were assessed at admission and again before discharge within a single, continuous hospitalization (**Figure 1**). Both groups were managed within the same orthopedic nursing unit by the same nursing team, and the nurse-to-patient ratio and overall staffing levels were comparable across the two enrollment periods; the principal difference between the groups was the content of the nursing protocol delivered rather than the number or composition of the nursing staff.

#### Inclusion criteria

- Thoracolumbar compression fracture confirmed by radiographic imaging (X-ray and/or computed tomography);
- Diagnosis of depressive disorder established in accordance with the Expert Consensus on Diagnosis and Treatment of Depressive Disorder by Integrated Chinese and Western Medicine (10) and confirmed by a Zung Self-Rating Depression Scale (SDS) standard score ≥= 53 points.
- Age ≥ 18 years;
- Patient and family members voluntarily signed an informed consent form.

#### Exclusion criteria

- Comorbid somatic disorders known to independently cause or exacerbate depressive symptoms (e.g., hypothyroidism, active malignancy, autoimmune disease);
- Comorbid psychiatric or psychological disorders other than depression (e.g., bipolar disorder, schizophrenia);
- Long-term use of dependence-forming substances;
- Pregnant or lactating women;
- Psychiatric symptoms induced by non-addictive substances.

#### Sample size

A total of 60 patients (30 per group) were enrolled. Based on a two-sided independent-samples t-test (α = 0.05, power = 0.80), a sample size of 30 per group provides adequate power to detect a between-group effect of Cohen’s d ≈ 0.74 or larger for each continuous outcome, which was considered the minimum clinically meaningful effect for the primary endpoints.

### Interventions

#### Control group — conventional nursing care

Patients in the control group received routine nursing care, which consisted of standard condition monitoring (vital signs, neurological status), health education regarding the fracture and its expected course, dietary guidance, and medication administration as prescribed.

#### Research group — comprehensive nursing intervention

In addition to the conventional care components described above, patients in the research group received a comprehensive, multidimensional nursing intervention comprising the following components:

Psychological care: Nursing staff closely monitored each patient’s psychological state and established a supportive nurse–patient relationship. Detailed explanations of the treatment plan, medications, and rehabilitation process were provided. Patients exhibiting marked negative affect received timely individualized counseling; when necessary, the attending physician was consulted for combined pharmacological and psychological management to help alleviate adverse psychological states and build confidence in recovery.

Environmental care: Wards were cleaned on a fixed schedule, with attention to adequate ventilation, natural lighting, and appropriate temperature and humidity. A humanistic color scheme with warm tones and indoor green plants was adopted, guided by the principle of naturalization, to create a comfortable, home-like atmosphere intended to convey the support of family and society.

Position nursing: Based on each patient’s specific fracture site, nursing staff assisted with optimal positioning. Bolsters were placed at limb–mattress contact points, and patients were helped to turn and reposition at regular intervals to maintain comfort and prevent pressure-related complications.

Pain care: Patients’ pain was assessed using the Visual Analogue Scale (VAS). After attentive listening, patients and families were informed of the pain-management plan. Physical measures (e.g., ice-pack application) were employed first; if these proved insufficient, patient-controlled analgesic pumps or analgesic medications were administered according to physician orders.

Health guidance: Each patient’s condition was individually evaluated, and a tailored rehabilitation plan was developed. Health-knowledge seminars were organized, supplemented by leaflets and educational videos covering both depression and fracture recovery. Emphasis was placed on helping patients recognize their psychological symptoms, understand their potential impact on recovery, and develop healthy strategies for emotional regulation.

Rehabilitation exercise: A comprehensive assessment of each patient’s physical capacity was performed, and an individualized rehabilitation plan was formulated. Patients were encouraged to begin rehabilitation exercises early after surgery, with exercise intensity and duration adjusted to individual tolerance levels.

Daily living care: Patients were provided access to leisure and recreational activities to cultivate positive interests and reinforce a constructive attitude toward life. These activities were chosen to be feasible for patients with restricted mobility and included low-intensity group board games and puzzles, simple handicrafts such as paper folding and drawing, supervised music-listening sessions, and the provision of books and magazines. They were organized and facilitated by the ward nursing staff, either at the bedside or in the patient lounge, generally in small groups of three to five patients, and were offered on most days of the week in sessions of approximately thirty to sixty minutes, scheduled around treatment, rehabilitation, and rest periods. Participation was entirely voluntary, and patients were free to join, leave, or decline any activity according to their physical condition and personal preference. Patients with favorable recovery trajectories were encouraged to share their experiences with peers, leveraging positive peer influence to help alleviate negative emotions across the ward.

**Figure 2:**
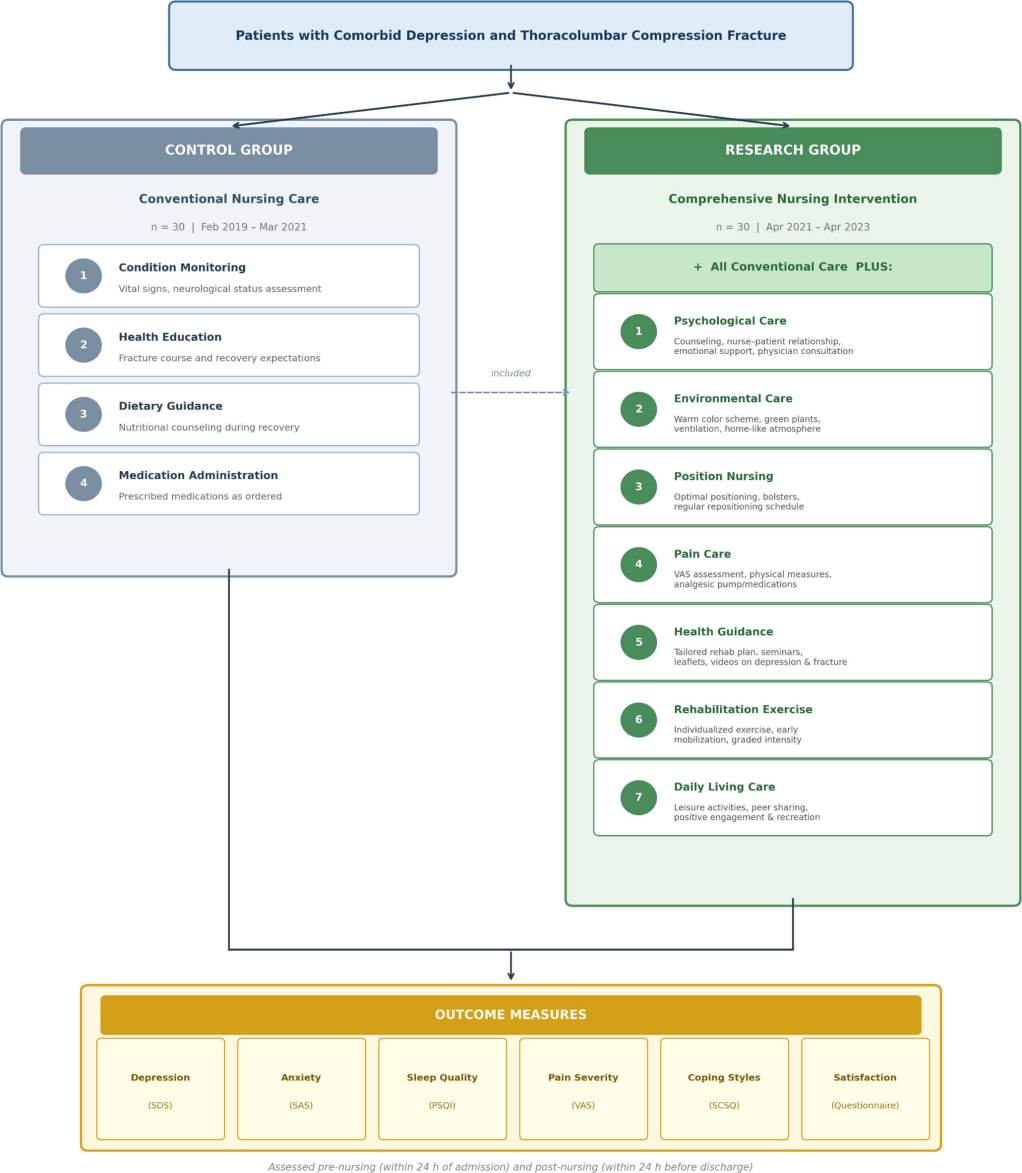
Framework of comprehensive nursing intervention compared with conventional nursing care, showing the seven additional intervention components and the six outcome domains assessed.

Intervention duration and fidelity: The comprehensive nursing intervention was delivered throughout each patient’s hospitalization. All nursing staff delivering the intervention received standardized training in the comprehensive nursing protocol before the study’s intervention period commenced. Conventional care components were identical across both groups. The components of both nursing interventions are summarized in **Figure 2**.

### Outcome measures

All outcomes were assessed at two time points: within 24 hours of admission (pre-nursing) and within 24 hours before discharge (post-nursing). To reduce assessment bias, outcome instruments were administered by trained research nurses who had no direct involvement in delivering either the conventional or comprehensive nursing intervention. Before the study commenced, all research nurses responsible for administering the instruments completed standardized training in the administration and scoring of each questionnaire to ensure consistency across assessors. Data were first recorded on standardized paper case-report forms at the bedside and subsequently entered into an electronic database. As a quality-control measure, completed forms were reviewed for completeness at the time of collection, any missing or ambiguous responses were clarified with the patient before the session concluded, and the electronic entries were independently cross-checked against the source forms to minimize transcription errors. For all four standardized self-report instruments (the SDS, SAS, PSQI, and SCSQ), the validated Chinese-language versions were used. These versions have been formally translated, culturally adapted, and shown to possess satisfactory reliability and validity in Chinese clinical populations, and the internationally established clinical cutoffs were applied without modification. The VAS is a non-verbal, language-independent measure and was used in its standard form. The corresponding validation references for each instrument are cited at first mention below.

### Psychological state (primary outcome)

The Zung Self-Rating Depression Scale (SDS) (11) and the Zung Self-Rating Anxiety Scale (SAS) (12) were used to assess depressive and anxious symptomatology, respectively. Both scales comprise 20 items scored on a four-point Likert scale (1 = a little of the time; 4 = most of the time). Raw scores are converted to standard scores (range 25–100) by multiplying by 1.25. An SDS standard score ≥ 53 indicates clinically significant depression; an SAS standard score ≥ 50 indicates clinically significant anxiety. Higher scores reflect greater symptom severity.

### Sleep quality

The Pittsburgh Sleep Quality Index (PSQI) (13) was used to evaluate sleep quality. The PSQI consists of 18 self-rated items contributing to seven component scores (subjective sleep quality, sleep latency, sleep duration, habitual sleep efficiency, sleep disturbance, use of sleep medication, and daytime dysfunction), each scored 0–3, yielding a global score of 0–21. A global score ≥ 7 indicates the presence of a sleep disorder; higher scores denote poorer sleep quality.

### Pain severity

The Visual Analogue Scale (VAS) (14) was used to quantify pain severity. Patients indicated their current pain intensity on a 10 cm horizontal line, where 0 cm represents no pain and 10 cm represents the worst imaginable pain.

### Coping styles

The Simplified Coping Style Questionnaire (SCSQ) (15, 16) was used to assess coping patterns. The SCSQ comprises 20 items divided into a positive coping subscale and a negative coping subscale, each scored on a four-point scale from 0 (“not adopted”) to 3 (“frequently adopted”). Higher positive coping scores indicate more adaptive strategies; higher negative coping scores indicate more maladaptive strategies.

### Nursing satisfaction

A self-developed nursing satisfaction questionnaire covering nursing content, ward environment, and staff attitude (total score 0–100) was administered at discharge. Scores of 91–100 were classified as “satisfied,” 66–90 as “generally satisfied,” and 0–65 as “dissatisfied.” Total satisfaction was defined as the combined proportion of “satisfied” and “generally satisfied” responses.

### Statistical analyses

All statistical analyses were performed using SPSS version 24.0 (IBM Corp., Armonk, NY, USA). The significance level was set at α = 0.05 (two-tailed) for all tests.

Continuous variables were evaluated for normality using the Shapiro–Wilk test, appropriate for the sample size of 30 per group. Normality was confirmed separately for each outcome variable. Homogeneity of variance was assessed for each outcome variable with Levene’s test for all between-group comparisons; where equal-variance assumptions were violated, Welch’s corrected t-test was applied. All continuous outcome variables satisfied both the normality and the homogeneity-of-variance assumptions and are therefore expressed as mean ± standard deviation (SD).

Baseline and post-nursing continuous outcomes were compared between the two groups using independent-samples t-tests (df = 58). Categorical variables (sex, nursing satisfaction categories) were compared using Pearson’s chi-square (χ²) test of independence. When any expected cell frequency fell below 5, Fisher’s exact test was used instead.

Pre-nursing versus post-nursing scores within each group were compared using paired-samples t-tests (df = 29), as the same patients were measured at both time points.

Cohen’s d was calculated for all between-group comparisons of continuous outcomes, using the pooled standard deviation as the denominator, and is reported with 95% confidence intervals (CIs). For categorical outcomes, the odds ratio (OR) with 95% CI was computed as the measure of effect size.

Seven between-group post-nursing comparisons were performed across the five outcome domains (SDS, SAS, PSQI, VAS, positive coping, negative coping, nursing satisfaction). To control the familywise error rate, the Holm–Bonferroni sequential correction was applied to all post-nursing between-group p-values. Count data are expressed as frequencies and percentages, n (%).

## Results

### Participant characteristics

A total of 60 patients were included in the analysis, with 30 in each group. Baseline demographic and clinical characteristics are presented in **Table 1**. The control group comprised 10 males (33.33%) and 20 females (66.67%), while the research group comprised 5 males (16.67%) and 25 females (83.33%). The between-group difference in sex distribution was not statistically significant (χ² test: df = 1, p = 0.136). Mean age was 68.53 ± 9.79 years in the control group and 69.87 ± 6.05 years in the research group (independent-samples t-test: df = 58, p = 0.526; Cohen’s d = 0.16, 95% CI [−0.34, 0.67]). The mean duration of depression was 10.23 ± 2.14 months in the control group and 10.33 ± 2.12 months in the research group (independent-samples t-test: df = 58, p = 0.856; Cohen’s d = 0.05, 95% CI [−0.46, 0.55]). None of the baseline comparisons reached statistical significance, confirming that the two groups were comparable at enrollment (**Table 1**).

**Table 1:** Baseline demographic and clinical characteristics of the two groups.

Factor	Category	Control group (n = 30)	Research group (n = 30)	Statistic	*p*
Sex, n (%)	Male	10 (33.33)	5 (16.67)	χ² = 2.222	0.136
	Female	20 (66.67)	25 (83.33)		
Age (mean ± SD, years)		68.53 ± 9.79	69.87 ± 6.05	t = 0.638	0.526
Depression duration (mean ± SD, months)		10.23 ± 2.14	10.33 ± 2.12	t = 0.182	0.856

Continuous data are presented as mean ± SD. Categorical data are presented as n (%). Between-group comparisons used independent-samples t-tests (age, depression duration) and the chi-square test (sex). SD, standard deviation.

### Psychological state

Pre-nursing SDS and SAS scores were comparable between groups, with no statistically significant differences (SDS: independent-samples t-test: df = 58, p = 0.907; SAS: independent-samples t-test: df = 58, p = 0.929), confirming equivalent baseline psychological status (**Table 2**).

**Table 2:** Comparison of SDS and SAS scores before and after nursing (mean ± SD, points).

Group	SDS				SAS			
	Pre-nursing	Post-nursing	*t*	*p*	Pre-nursing	Post-nursing	*t*	*p*
Control (n = 30)	85.10 ± 5.63	70.17 ± 7.80	10.40	< 0.001	85.20 ± 5.65	67.50 ± 7.55	12.62	< 0.001
Research (n = 30)	84.93 ± 5.58	56.83 ± 8.45	18.46	< 0.001	85.07 ± 5.61	54.30 ± 8.10	19.87	< 0.001
*t* (between)	0.117	6.35			0.089	6.56		
*p* (between)	0.907	< 0.001			0.929	< 0.001		

Within-group comparisons (pre- vs. post-nursing) used paired-samples t-tests (df = 29). Between-group comparisons (control vs. research) used independent-samples t-tests (df = 58). SDS, Zung Self-Rating Depression Scale (clinical threshold ≥ 53); SAS, Zung Self-Rating Anxiety Scale (clinical threshold ≥ 50). Post-nursing between-group effect sizes: SDS Cohen’s d = 1.64 (95% CI: 1.04 to 2.23); SAS Cohen’s d = 1.69 (95% CI: 1.08 to 2.29). Both p-values survived Holm–Bonferroni correction.

Both groups demonstrated significant reductions in depressive and anxiety symptomatology following their respective nursing interventions. In the control group, the mean SDS score decreased from 85.10 ± 5.63 to 70.17 ± 7.80 (paired t-test: df = 29, p < 0.001), and the mean SAS score decreased from 85.20 ± 5.65 to 67.50 ± 7.55 (paired t-test: df = 29, p < 0.001). In the research group, the reductions were markedly greater: the mean SDS score decreased from 84.93 ± 5.58 to 56.83 ± 8.45 (paired t-test: df = 29, p < 0.001), and the mean SAS score decreased from 85.07 ± 5.61 to 54.30 ± 8.10 (paired t-test: df = 29, p < 0.001).

Critically, the research group demonstrated significantly lower post-nursing scores than the control group for both measures. The mean post-nursing SDS score was 13.34 points lower in the research group (independent-samples t-test: df = 58, p < 0.001; Cohen’s d = 1.64, 95% CI [1.04, 2.23]), and the mean post-nursing SAS score was 13.20 points lower (independent-samples t-test: df = 58, p < 0.001; Cohen’s d = 1.69, 95% CI [1.08, 2.29]). Notably, the post-nursing SDS score in the control group (70.17 ± 7.80) remained well above the clinical threshold for depression (≥ 53), whereas the mean in the research group (56.83 ± 8.45) approached the threshold, suggesting that a substantial proportion of patients in the research group achieved clinically meaningful improvement. A parallel pattern was observed for SAS relative to the anxiety threshold (≥ 50), with the research group’s post-nursing mean (54.30 ± 8.10) approaching the clinical cutoff (**Table 2**). Both comparisons remained significant after Holm–Bonferroni correction (adjusted α_1_ = 0.007, adjusted α_2_ = 0.008).

### Sleep quality

Pre-nursing PSQI scores were comparable between groups (control: 17.23 ± 1.99; research: 17.40 ± 1.94; independent-samples t-test: df = 58, p = 0.739), indicating equivalent baseline sleep disturbance (**Table 3**).

**Table 3:** Comparison of PSQI scores before and after nursing (mean ± SD, points).

Group	Pre-nursing	Post-nursing	*t*	*p*
Control (n = 30)	17.23 ± 1.99	12.47 ± 2.90	9.33	< 0.001
Research (n = 30)	17.40 ± 1.94	7.83 ± 2.55	18.78	< 0.001
*t* (between)	0.335	6.57		
*p* (between)	0.739	< 0.001		

Within-group comparisons used paired-samples t-tests (df = 29). Between-group comparisons used independent-samples t-tests (df = 58). PSQI, Pittsburgh Sleep Quality Index (clinical threshold ≥ 7; range 0–21; higher scores indicate poorer sleep quality). Post-nursing between-group Cohen’s d = 1.70 (95% CI: 1.08 to 2.31). The p-value survived Holm–Bonferroni correction.

Following intervention, both groups exhibited significant improvements. The mean PSQI score in the control group declined to 12.47 ± 2.90 (paired t-test: df = 29, p < 0.001), and in the research group it declined to 7.83 ± 2.55 (paired t-test: df = 29, p < 0.001). The post-nursing between-group comparison revealed a significantly lower PSQI score in the research group (independent-samples t-test: df = 58, p < 0.001; Cohen’s d = 1.70, 95% CI [1.08, 2.31]). Although both groups remained above the clinical sleep-disorder threshold (≥ 7) at discharge, the research group’s mean score (7.83) was substantially closer to normalization than the control group’s score (12.47), indicating a more favorable trajectory toward restored sleep quality (**Table 3**). This comparison remained significant after Holm–Bonferroni correction (adjusted α_3_= 0.010).

### Pain severity

Pre-nursing VAS scores were comparable between groups (control: 7.13 ± 0.51; research: 7.20 ± 0.43; independent-samples t-test: df = 58, p = 0.568), reflecting similar baseline pain intensity in the moderate-to-severe range (**Table 4**).

**Table 4:** Comparison of VAS scores before and after nursing (mean ± SD, points).

Group	Pre-nursing	Post-nursing	*t*	*p*
Control (n = 30)	7.13 ± 0.51	4.93 ± 1.25	9.37	< 0.001
Research (n = 30)	7.20 ± 0.43	3.17 ± 1.10	20.02	< 0.001
*t* (between)	0.574	5.79		
*p* (between)	0.568	< 0.001		

Within-group comparisons used paired-samples t-tests (df = 29). Between-group comparisons used independent-samples t-tests (df = 58). VAS, Visual Analogue Scale (range 0–10; higher scores indicate greater pain severity). Post-nursing between-group Cohen’s d = 1.50 (95% CI: 0.90 to 2.08). The p-value survived Holm–Bonferroni correction.

Both groups showed significant reductions in pain following intervention. The mean VAS score in the control group decreased to 4.93 ± 1.25 (paired t-test: df = 29, p < 0.001), while in the research group it decreased to 3.17 ± 1.10 (paired t-test: df = 29, p < 0.001). The research group’s post-nursing VAS score was significantly lower than that of the control group (independent-samples t-test: df = 58, p < 0.001; Cohen’s d = 1.50, 95% CI [0.90, 2.08]), corresponding to a clinically meaningful difference of 1.76 points on the 10 cm scale. This indicates that the multimodal pain-management component of the comprehensive intervention conferred additional analgesic benefit beyond conventional care (**Table 4**). This comparison remained significant after Holm–Bonferroni correction (adjusted α_4_ = 0.013).

### Coping styles

Pre-nursing positive and negative coping scores were comparable between groups (positive: independent-samples t-test: df = 58, p = 0.717; negative: independent-samples t-test: df = 58, p = 0.914), confirming equivalent baseline coping patterns (**Table 5**).

**Table 5:** Comparison of Positive and negative coping style scores before and after nursing (mean ± SD, points).

Group	Positive coping				Negative coping			
	Pre-nursing	Post-nursing	*t*	*p*	Pre-nursing	Post-nursing	*t*	*p*
Control (n = 30)	10.23 ± 1.52	16.07 ± 3.85	9.13	< 0.001	16.23 ± 2.50	11.27 ± 2.65	9.70	< 0.001
Research (n = 30)	10.37 ± 1.45	21.53 ± 4.20	16.08	< 0.001	16.30 ± 2.47	7.83 ± 2.40	17.84	< 0.001
*t* (between)	0.365	5.25			0.109	5.27		
*p* (between)	0.717	< 0.001			0.914	< 0.001		

Within-group comparisons used paired-samples t-tests (df = 29). Between-group comparisons used independent-samples t-tests (df = 58). SCSQ, Simplified Coping Style Questionnaire. Higher positive coping scores indicate more adaptive strategies; higher negative coping scores indicate more maladaptive strategies. Post-nursing between-group effect sizes: positive coping Cohen’s d = 1.35 (95% CI: 0.78 to 1.93); negative coping Cohen’s d = 1.36 (95% CI: 0.79 to 1.94). Both p-values survived Holm–Bonferroni correction.

After intervention, positive coping scores increased and negative coping scores decreased in both groups. In the control group, the positive coping score rose from 10.23 ± 1.52 to 16.07 ± 3.85 (paired t-test: df = 29, p < 0.001) and the negative coping score fell from 16.23 ± 2.50 to 11.27 ± 2.65 (paired t-test: df = 29, p < 0.001). In the research group, the positive coping score rose from 10.37 ± 1.45 to 21.53 ± 4.20 (paired t-test: df = 29, p < 0.001) and the negative coping score fell from 16.30 ± 2.47 to 7.83 ± 2.40 (paired t-test: df = 29, p < 0.001).

Between-group comparisons at the post-nursing time point revealed significantly higher positive coping scores (independent-samples t-test: df = 58, p < 0.001; Cohen’s d = 1.35, 95% CI [0.78, 1.93]) and significantly lower negative coping scores (independent-samples t-test: df = 58, p < 0.001; Cohen’s d = 1.36, 95% CI [0.79, 1.94]) in the research group relative to the control group. These findings indicate that the comprehensive nursing intervention was associated with a substantially more pronounced shift from maladaptive toward adaptive coping strategies than conventional care (**Table 5**). Both coping comparisons remained significant after Holm–Bonferroni correction (adjusted α_5_ = 0.017, adjusted α_6_ = 0.025).

### Nursing satisfaction

Total nursing satisfaction (defined as “satisfied” plus “generally satisfied”) was 96.67% (29/30) in the research group compared with 66.67% (20/30) in the control group (Fisher’s exact test: p = 0.003; OR = 14.50, 95% CI [1.72, 122.24]) (**Table 6**). Within the research group, 76.67% of patients rated their care as “satisfied” (the highest category), compared with only 16.67% in the control group. This comparison survived Holm–Bonferroni correction (adjusted α_7_ = 0.050). The wide 95% CI of the odds ratio reflects the small cell count in the research-group “dissatisfied” category (n = 1) and indicates imprecision in the effect-size point estimate, which should be interpreted with caution.

**Table 6:** Comparison of nursing satisfaction between the two groups, n (%).

Group	Satisfied	Generally satisfied	Dissatisfied	Total satisfaction
Control (n = 30)	5 (16.67)	15 (50.00)	10 (33.33)	20 (66.67)
Research (n = 30)	23 (76.67)	6 (20.00)	1 (3.33)	29 (96.67)
				Fisher’s exact p = 0.003

Total satisfaction = Satisfied + Generally satisfied. Between-group comparison used Fisher’s exact test (applied because one expected cell frequency was below 6). OR = 14.50 (95% CI: 1.72 to 122.24). The wide confidence interval reflects the small cell count in the research-group “dissatisfied” category (n = 1).

## Discussion

The present study evaluated a comprehensive nursing intervention for patients with comorbid depression and thoracolumbar compression fracture. The results demonstrated that, compared with conventional care, the comprehensive intervention was associated with significantly greater improvements in depressive and anxiety symptoms, sleep quality, pain severity, and coping styles, along with higher nursing satisfaction. All between-group differences remained statistically significant after Holm–Bonferroni correction for multiple comparisons, and the corresponding effect sizes (Cohen’s d = 1.35–1.70) indicate large and clinically meaningful effects.

Both groups showed significant pre-to-post reductions in SDS and SAS scores, indicating that even conventional nursing confers some psychological benefit during hospitalization. However, the research group showed substantially greater improvement (SDS: d = 1.64; SAS: d = 1.69). The post-nursing SDS score in the research group (56.83 ± 8.45) approached the clinical threshold for depression (≥ 53), suggesting that a meaningful proportion of patients achieved clinically relevant improvement, whereas the control group’s mean (70.17 ± 7.80) remained well above this cutoff. These findings suggest that the psychological care, health guidance, and cognitive-behavioral components of the comprehensive intervention were effective in addressing the emotional needs of this population. The structured approach, combining nurse-led counseling with physician collaboration for patients exhibiting severe negative affect, likely contributed to the observed improvements by helping patients develop realistic expectations for recovery and effective emotional regulation strategies (17, 18). Furthermore, regular behavioral and cognitive health education sessions may have helped patients understand the relationship between their psychological state and physical recovery, thereby reducing feelings of helplessness (19).

Sleep disturbance is common among hospitalized patients with fractures, and is further exacerbated by comorbid depression. Both environmental factors (noise, unfamiliar surroundings) and disease-related factors (pain, psychological distress) contribute to disrupted sleep architecture and impaired regulation of the sleep–wake cycle (20–22). In this study, PSQI scores improved in both groups, but the research group showed a significantly greater reduction (d = 1.70). Although the research group’s mean post-nursing score (7.83) remained near the clinical threshold for sleep disorder (≥ 7), this represents a substantial improvement from the severe baseline disturbance (17.40) and a notably better outcome than the control group’s post-nursing score (12.47). The ward environmental modifications, including optimized lighting, temperature control, and a calming aesthetic, along with structured rehabilitation exercise, likely contributed to improved sleep by reducing environmental stressors and promoting physical fatigue (23, 24).

Pain is a central concern in the rehabilitation of thoracolumbar fracture patients and is a recognized driver of depressive symptoms (4). Both groups experienced significant pain reduction, but the research group achieved a meaningfully lower VAS score (3.17 vs. 4.93; d = 1.50), corresponding to an additional 1.76-point improvement on the 10 cm scale. This difference exceeds the commonly accepted minimum clinically important difference for VAS in musculoskeletal pain (approximately 1.0–1.5 cm) (25, 26), supporting the clinical relevance of the stepped pain-management approach used in the comprehensive intervention, progressing from physical measures to patient-controlled analgesia as guided by individualized assessment.

Maladaptive coping has been linked to poorer outcomes in patients with chronic pain and depression (27). In this study, the comprehensive intervention was associated with a greater increase in positive coping scores (d = 1.35) and a greater decrease in negative coping scores (d = 1.36). This shift likely reflects the combined effect of health guidance sessions, which helped patients understand their condition and develop constructive emotional outlets, and daily living care activities designed to foster positive interests and peer support. The individualized, stepwise nature of the intervention, with regular reassessment and adjustment, may have been particularly important in building patients’ confidence and self-efficacy.

Total nursing satisfaction was significantly higher in the research group (96.67% vs. 66.67%; OR = 14.50). This finding aligns with the broader premise that nursing care addressing psychological needs, comfort, and health education in addition to disease management yields higher patient satisfaction (28, 29). The wide confidence interval of the odds ratio (1.72–122.24), however, reflects the small sample size, and the magnitude of this effect should be interpreted with caution.

Several limitations should be considered when interpreting these findings. First, the non-concurrent historical-control design means that the two groups were treated in different time periods, and unmeasured period effects (such as changes in clinical staffing, concurrent treatment protocols, or patient demographics) cannot be excluded as confounding factors. Second, the sample size was small (n = 30 per group), which limits statistical precision, as reflected in the wide confidence interval for the satisfaction odds ratio, and limits the generalizability of the findings. Third, assessments were limited to the hospitalization period; longer-term follow-up would be needed to determine whether the observed benefits persist after discharge. Fourth, although outcome assessors were not directly involved in delivering the intervention, formal blinding of assessors to group allocation was not implemented, introducing the possibility of assessment bias. Fifth, the nursing satisfaction questionnaire was self-developed and has not undergone formal psychometric validation; its reliability and validity remain unestablished. Sixth, because both conditions contribute to the measured outcomes, the study cannot fully disentangle the effects of the intervention on depression-related symptoms from those on fracture-related pain and functional limitation. Seventh, the additional resources required to deliver the comprehensive intervention, including nursing time and the costs of environmental modification and recreational provision, were not formally recorded, and a pharmacoeconomic evaluation of its cost-effectiveness and real-world sustainability could therefore not be undertaken. Eighth, although analgesic and antidepressant medications were prescribed according to standardized institutional protocols in both groups, detailed pharmacological data, including the specific agents used, their doses, and any associated adverse effects, were not systematically collected, so the possibility that between-group differences in medication contributed to the observed outcomes cannot be entirely excluded. Future research should address these limitations by employing a prospective randomized controlled design, larger sample sizes, validated satisfaction instruments, blinded outcome assessment, and extended follow-up to evaluate the durability of the intervention’s effects. In addition, future studies should incorporate a prospective economic evaluation of cost-effectiveness, together with detailed documentation of concurrent pharmacological treatment, to support real-world implementation and to further clarify the mechanisms underlying the observed benefits.

## Conclusion

In summary, the comprehensive nursing intervention for patients with comorbid depression and thoracolumbar compression fracture was associated with significantly greater improvements in depressive symptoms, anxiety, sleep quality, pain severity, and coping styles compared with conventional care, alongside higher nursing satisfaction. These results support the integration of psychological, environmental, and rehabilitative components into routine nursing care for this patient population. From a clinical standpoint, the findings suggest that embedding structured psychological support, environmental optimization, and individualized pain and rehabilitation management within standard ward nursing is both feasible and potentially beneficial, and that nurses are well positioned to deliver such integrated care to address the intertwined emotional and physical needs of this population. However, the non-randomized design and small sample size limit the strength of these conclusions. Confirmation through larger, multicenter, prospective controlled studies with extended follow-up and validated outcome instruments is warranted before definitive clinical recommendations can be made.

## Data Availability

The raw data supporting the conclusions of this article will be made available by the authors, without undue reservation.
